# Complete Neuron Reconstruction Based on Branch Confidence

**DOI:** 10.3390/brainsci14040396

**Published:** 2024-04-19

**Authors:** Ying Zeng, Yimin Wang

**Affiliations:** 1 School of Computer Science and Technology, Shanghai University, Shanghai 200444, China; zenyints@shu.edu.cn; 2 Guangdong Institute of Intelligence Science and Technology, Zhuhai 519031, China

**Keywords:** neuron reconstruction, analysis of neuronal features, confidence of branch, Markov chain, image processing

## Abstract

In the past few years, significant advancements in microscopic imaging technology have led to the production of numerous high-resolution images capturing brain neurons at the micrometer scale. The reconstructed structure of neurons from neuronal images can serve as a valuable reference for research in brain diseases and neuroscience. Currently, there lacks an accurate and efficient method for neuron reconstruction. Manual reconstruction remains the primary approach, offering high accuracy but requiring significant time investment. While some automatic reconstruction methods are faster, they often sacrifice accuracy and cannot be directly relied upon. Therefore, the primary goal of this paper is to develop a neuron reconstruction tool that is both efficient and accurate. The tool aids users in reconstructing complete neurons by calculating the confidence of branches during the reconstruction process. The method models the neuron reconstruction as multiple Markov chains, and calculates the confidence of the connections between branches by simulating the reconstruction artifacts in the results. Users iteratively modify low-confidence branches to ensure precise and efficient neuron reconstruction. Experiments on both the publicly accessible BigNeuron dataset and a self-created Whole-Brain dataset demonstrate that the tool achieves high accuracy similar to manual reconstruction, while significantly reducing reconstruction time.

## 1. Introduction

Neurons constitute the fundamental units of the brain’s nervous system. A comprehensive insight of their morphological structure is crucial for studying brain function and diseases [1,2]. For instance, reduced dendritic branching has been detected in the CA1 and CA4 subregions of the hippocampus in autism patients, while decreased lengths of dendritic branches in both apical and basal regions of the CA1a and CA1b subregions have been observed in Alzheimer’s disease patients [3].

In recent years, advanced fluorescence imaging platforms capable of high-throughput and high-resolution have been developed, such as fluorescence micro-optical sectioning tomography (fMOST) [4,5] and light-sheet fluorescence microscopy (LSFM) [6,7]. These technologies have facilitated the production of numerous large-scale images of neurons. Due to imaging technology constraints and uneven fluorescent labeling of neurons, images often suffer from high levels of noise and low signal-to-noise ratios. Consequently, analyzing neuron structure becomes challenging. Neuron reconstruction aims to extract the morphological structure of neurons from these images. Presently, there is no method for neuron reconstruction that is entirely accurate due to these constraints. Therefore, manual reconstruction remains essential for achieving highly precise results.

To facilitate users to manually reconstruct neurons, various neuron reconstruction tools have been created, including Neurolucida [8], Vaa3D [9], Janelia Workstation [10], and so on. Although the manual reconstruction of neurons achieved high accuracy, the more complex the neuron morphology, the higher the time cost. As a rough estimate, a complex cortical neuron typically requires approximately 1–3 weeks to completely reconstruct [11].

Driven by the 2010 DIADEM competition, scientists have proposed many automatic tracing algorithms [12], such as the APP2 method, which prunes over-reconstruction according to a hierarchical strategy prioritizing long segments [13], the MOST method for centerline extraction based on initial seed points [14], and the ENT method, which refers to ensemble learning for neuron reconstruction [15], all of which aim to improve the efficiency of neuron reconstruction. In recent years, scientists have introduced deep learning methods into the neuron reconstruction. The 3D U-Net Plus method [16] improved 3D U-Net network to segment tangled neuronal image; the UltraNPR method [17] segmented large-scale neuron cluster images based on progressive learning; SPE-DNR [18] reconstructed neurons by detecting critical points. These methods have shown promising results in neuronal image segmentation and critical point extraction. However, due to the varying strategies and models used by automatic reconstruction algorithms, as well as the differences in images processed by the algorithms, their performance is not satisfactory in practical applications, and the reconstruction cannot be directly utilized.

Unlike direct automatic tracing reconstruction algorithms, probabilistic tracing reconstructs neurons in an uncertain manner, involving the calculation of confidence for seed points or segments. Radojeić et al. introduced a neuron tracing method that utilizes probability hypothesis density (PHD) filtering to assign tubular value confidence to seed points [19]. Similarly, Athey et al. tackled the neuron tracing problem by employing a hidden Markov model, connecting the two neuronal segments with the highest connection confidence, and deriving the final probability reconstruction [20]. Inspired by the concept of probabilistic tracing, this paper implements confidence calculation on neuron reconstruction. High-confidence branches are retained, while low-confidence branches are presented to the users for modification, aiming to achieve efficient and accurate neuron reconstruction results.

**Figure 1:**
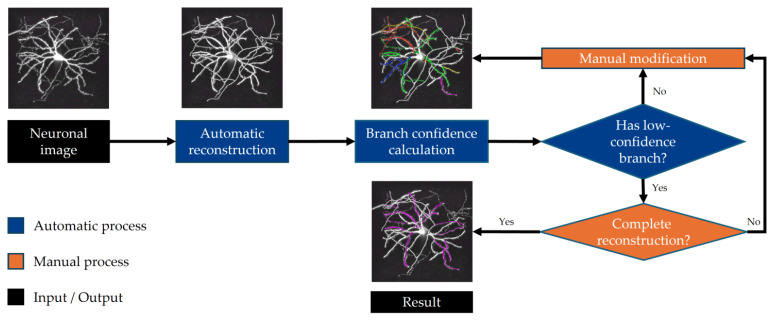
A flowchart of the proposed reconstruction process. Branches affected by reconstruction artifacts are highlighted with different colors. In particular, white indicates high-confidence branches, while fuchsine denotes user-confirmed correct branches.

Manual neuron reconstruction is known for its precision but requires a significant time investment. On the other hand, automatic reconstruction offers a quick solution but often sacrifices accuracy. Therefore, this paper attempts to develop a neuron reconstruction tool with both accuracy and efficiency. In this paper, we develop a tool for detecting reconstruction artifacts based on branch confidence calculation, which can assist users to reconstruct complete neurons. As shown in Figure 1, the tool provides users with feedback on reconstruction artifacts detected in the automatic reconstruction through various marking techniques, facilitating modification operations. By iteratively addressing low-confidence branches via a ‘detection–modification’ approach, users can achieve more accurate neuron reconstructions.

In summary, the major contributions of this paper are as follows:

(1) We propose an accurate and efficient neuron reconstruction process. By embedding the necessary manual modification process in the automatic reconstruction, the reconstruction accuracy is improved while reducing the time required for direct manual reconstruction.

(2) We design a method for detecting reconstruction artifacts, which serves as a valuable reference for other studies in neuron reconstruction. Leveraging the distinct features of neuronal branches, we employed Markov chain modeling to analyze the results of neuron reconstruction. Within this framework, reconstruction artifacts are simulated, facilitating their detection and prompting users for necessary modifications.

(3) We evaluated the performance of this neuron reconstruction tool in two challenging 3D neuronal image datasets. The experimental results show that our method can achieve accurate and rapid neuron reconstruction.

## 2. Materials and Methods

### 2.1. Artifacts of Neuron Reconstruction

**Figure 2:**
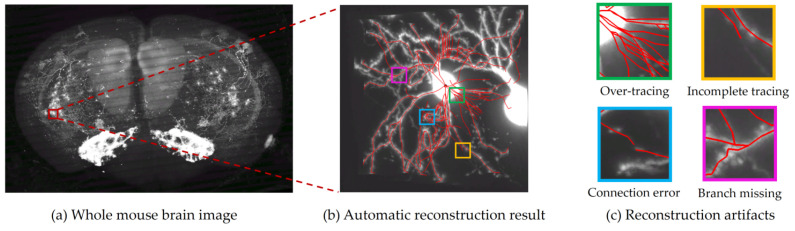
Automatic reconstruction of captured whole-brain neuron images.

Due to the uneven distribution of fluorescent labels in neurons and the limitation of optical microscopy imaging technology, the neuronal image often exhibits issues such as noise interference and voxel loss. Particularly at the whole-brain level, where neurons are densely intertwined, these complexities pose significant challenges for automatic reconstruction algorithms. As shown in Figure 2, a neuron is selected for automatic reconstruction under a whole-brain neuronal image of a mouse. The neuronal image exhibits noise and interference from other neuronal branches. When utilizing the traditional APP2 algorithm for automatic reconstruction, the result reveals unsatisfactory output, characterized by numerous artifacts. According to their different morphological characteristics, these artifacts can be divided into four categories: over-tracing, incomplete tracing, connection error, and branch missing. According to the range of the influence of artifacts on the branch, it can be broadly classified as global artifacts or local artifacts. In particular, some artifacts existing in parent branch will be directly inherited by the current branch, which can be called artifacts having transitivity.

Over-tracing occurs when an automatic algorithm traces redundant neuronal branches, usually due to image noise, and this artifact exhibits transitivity. Connection errors arise when the algorithm’s reconstruction result deviates from the typical pattern of neuronal growth, caused by connections to interfering branches, and this artifact also displays transitivity. Branch missing refers to cases where the automatic algorithm misses or fails to trace a sub-branch, either due to image noise or voxel loss, without transitivity in this artifact. Incomplete tracing denotes situation where the automatic algorithm stops tracing at positions that still require tracing, possibly due to voxel loss, leading to gaps in the branches without transitivity in this artifact. Incomplete tracing is a special type of branch missing that takes place at the end of a branch.

The four artifacts mentioned above have differing implications for branches. Over-tracing and connection errors within branches are categorized as global artifacts, while branch missing, incomplete tracing, and connection errors between branches pertain to local artifacts. Specifically, connection errors are categorized into two types: internal connection error, which occurs within a branch itself, indicating that the branch is connected to another interfering branch and results in incorrect reconstruction; and external connection error, which refers to a fragmentary connection error between two reconstructed branches. These two types of artifacts exist in distinct locations and need to be explained separately.

These reconstruction artifacts significantly reduce the accuracy of automatic reconstruction results, making them impractical for direct utilization and requiring manual adjustments by users. Especially, during the modification phase, users are compelled to evaluate each branch for four types of artifacts, resulting in a substantial consumption of manual labor. This paper aims to detect these four types of reconstruction artifacts in an automatic way. Users just need to adjust the low-confidence branches based on the tool’s hints, evaluating the detection results only once, so as to reduce the reconstruction time.

### 2.2. Common Features of Neuronal Branch

To identify artifacts in neuron reconstruction, understanding accurately reconstructed branch features is crucial. Neuronal branches have a tree-like structure, with a child branch sharing similar properties to its parent branch. They typically exhibit image features, like intensity mean and standard deviation, which reflect intensity information within the neuronal image. Meanwhile, for the structural features like angle between parent and child branches, reflects the morphological aspects of branches in the overall reconstruction. These features play an important role in detecting and analyzing potential artifacts in neuron reconstruction.

**Figure 3:**
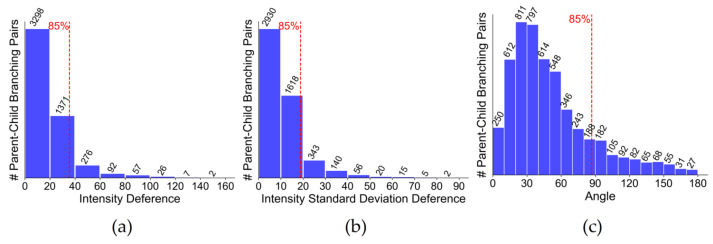
Statistical analysis of neuronal branches’ common features. The x-axis represents specific feature values, while the y-axis indicates the number of parent-child branch pairs corresponding to each feature value. (**a**) Examining intensity differences between parent–child branching pairs in the mouse brain. (**b**) Exploring intensity standard deviation differences among such pairs. (**c**) Investigating the distribution of the angles between parent–child branching pairs. The red dotted line divides the feature distribution, with the portion preceding it representing 85% of the total distribution.

Figure 3 shows the common features of branches in mouse neurons. The dataset is composed of 20 complete neurons manually reconstructed from whole-brain neuronal images of 7 different mice, and the neurons in 29 regions are extracted and divided into 5190 pairs of parent–child branches for feature statistics. In order to emphasize the similarity between parent and child branches, we calculate the differences in their features using three statistical metrics: intensity difference, intensity standard deviation difference, and angle. Branching pairs exhibiting neuronal features in more than 85% of cases are termed as the common features of neuronal branches. Figure 3 reveals that the intensity difference and intensity standard deviation difference of neuronal branches have similar distributions: as the value of feature surpasses a specific threshold, there is a sharp decrease in the count of branching pairs. This indicates that these two features can effectively capture the image features of neuron reconstruction. Moreover, when the intensity difference or the intensity standard deviation difference decreases, the number of parent–child branching pairs increases. This supports the concept that there is a similarity in intensity between parent and child branches. Finally, angle between parent and child branches is less than 90 degrees, which is aligned with the growth pattern of neuronal branches, and the probability of reverse growth is reduced. Therefore, this paper uses these three features as evaluation factors for artifact detection.

### 2.3. Model Designation

The Markov chain refers to a series of random events, where the output of each event depends only on the preceding event’s state, independent of previous events. In other words, the Markov chain has the memoryless property, where the current state is solely influenced by the preceding state, a characteristic known as Markov property.

**Figure 4:**
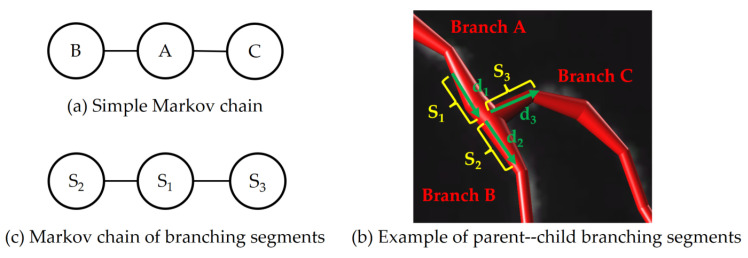
Markov chain in neuron reconstruction.

As shown in Figure 4a, assuming state *B* and state *C* are any two points in the undirected graph without direct edge connection, the probability of transferring from state *B* to state *C* is independent of state *B*, and this is expressed mathematically as follows:

(1) $$P\left(X_{C}|X_{B},X_{A}\right)=P\left(X_{C}|X_{A}\right)$$

Because of the tree-like structure of neuronal branches, the growth trend of the current branch is directly influenced by its parent branch, with no dependence on other branches for its growth. This property aligns neuronal branching pair with the Markov property and allows for its design using Markov chains.

In this paper, inspired by the work [20], we design Markov chains suitable for parent–child branching pairs of neurons. In the work [20], numerous small fragments were directly extracted from neuronal images. The connection confidence between these fragments was computed using hidden Markov random fields, enabling the neuron tracing with high confidence based on probability. In contrast to [20], this paper partitions the reconstructed neuron structure into several pairs of parent–child branching segments, as illustrated in Figure 4b. Beginning from the branching point, fixed-length and direction branching segments are, respectively, extracted from the parent–child branching pair. The direction of these branching segments is indicated by arrows. According to the context above, it is inferred that the two segments exhibit Markov property. Consequently, the reconstruction can be considered as the integration of several simple Markov chains, as depicted in Figure 4c. In this paper, the connection probabilities of parent–child branching segments are calculated by simulating various artifacts generated during reconstruction. And the branch confidence calculation is categorized into two categories based on the range of the artifacts’ influence on the branch, specifically denoted as:

(2) $$Confidence\left(Branch\;B\right)=\min(P_{G}\left(Branch\;B\right),P_{L}\left(Branch\;B\right))$$

where, $P_{G}$ refers to the probability that the parent–child branching pair is connected under the influence of a global artifact, and $P_{L}$ refers to the probability that the parent–child segment pair is connected under the influence of a local artifact. The definition is as follows:

(3) $$P_{G}\left(Branch\;B\right)=P\left(Branch\;B|Branch\;A\right)$$

(4) $$P_{L}\left(Branch\;B\right)=\sum_{b}^{Branch\;B}Confidence\left(b\right)$$

here, *b* is one of the segments of *Branch B*.

For the calculation of probability, the energy function U(·) is designed to calculate the energy required for the connection between branches. The greater the energy, the lower the probability of the connection. Taking BranchA and BranchB as an example, the connection probability is calculated as follows:

(5) $$P\left(Branch\;B|Branch\;A\right)=1-\frac{U(Branch\;A,Branch\;B)}{Z(Branch\;A)}$$

(6) $$Z\left(Branch\;A\right)=\sum_{s\in S}U\left(Branch\;A,s\right),\;S=\left\{s|0<dist\left(Branch\;A,s\right)<T_{d}\cup Branch\;B\right\}$$

here, Z(BranchA) calculates the total energy demand for all branches within set *S* to be connected to BranchA. Set *S* encompasses neighboring branches that potentially connect to BranchA. dist(·) calculates the nearest distance between two branching segment endpoints, and $T_{d}$ represents the distance threshold. At last, the current branch is added to the set *S*, considering the component of the current connection in the total potential connection.

To derive the energy function, we incorporate three common features of a neuronal branch, and the design is as follows:

(7) $$U\left(Branch\;A,Branch\;B\right)=\left\{\begin{array}{c} \alpha \Delta I+\beta \Delta \sigma ,\;\theta <90\\ \infty ,\;\theta \geq 90 \end{array}\right.$$

(8) $$\Delta I=\left|I\left(S_{1}\right)-I\left(S_{2}\right)\right|$$

(9) $$\Delta \sigma =\left|\sigma \left(S_{1}\right)-\sigma \left(S_{2}\right)\right|$$

(10) $$\cos\theta =\cos\langle d_{1},d_{2}\rangle$$

where ΔI refers to the average intensity difference corresponding to BranchA and BranchB in the image, while Δσ is the intensity standard deviation difference corresponding to the two branches in the image. The angle between the two branches is θ. Hyperparameters α and β regulate the importance assigned to the image features. Additionally, θ<90 is assumed as the prior knowledge for neuron reconstruction. If the angle of the parent and child branches is greater than 90 degrees, the connection energy is set to infinity. This indicates that it is impossible to establish a connection in this case, and promotes users to check and confirm. Branches with confidence below a specified threshold are marked as having low confidence, prompting user intervention for modifications.

### 2.4. Data Preprocessing and Skeleton Branch Extraction

For the purpose of reconstructing a complete neuron, it is essential to accurately identify each branch in the neuronal image. Nonetheless, the background intensity across the whole-brain neuronal image is not uniform, and there are neuronal branches with weak signals that may be easily ignored during reconstruction [21]. In this paper, we employ the derivative truncated gamma transformation (DTGT) method [22] to enhance the weak signals in branches, thereby ensuring the integrity of the reconstruction.

**Figure 5:**
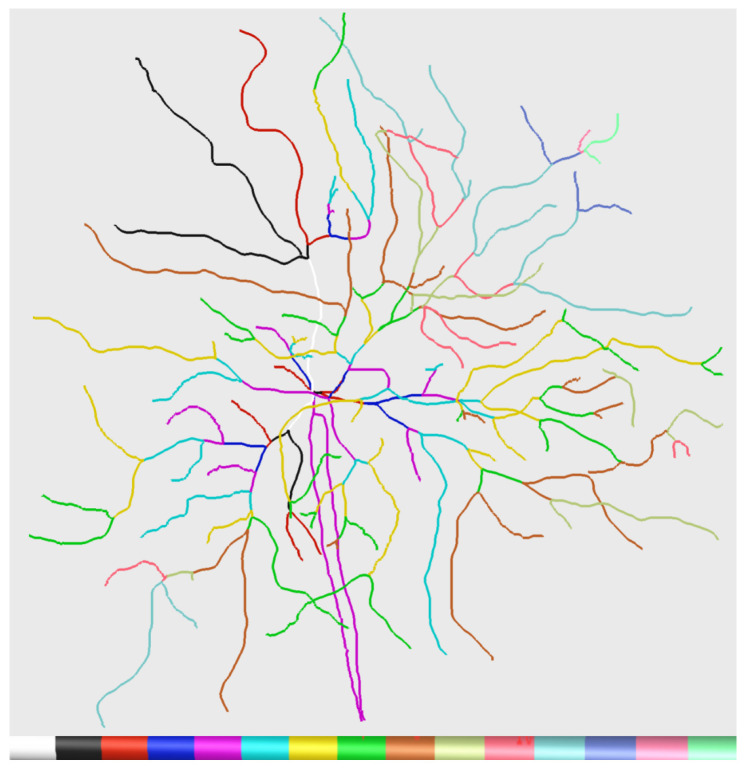
Neuronal branches hierarchical display. Branches are color-coded based on different reconstruction types in Vaa3D, indicating various hierarchical levels. The white value on the far left corresponds to 0, with levels increasing from left to right.

The proposed tool conducts confidence calculation for each branch. Given the transitivity of certain artifacts, the reconstructed neuronal branches should be extended from soma to calculate confidence, so it is necessary to divide the reconstructed neuronal branches based on the parent–child hierarchy within the tree structure. As depicted in Figure 5, branches sharing the same hierarchy are color-coded uniformly.

**Figure 6:**
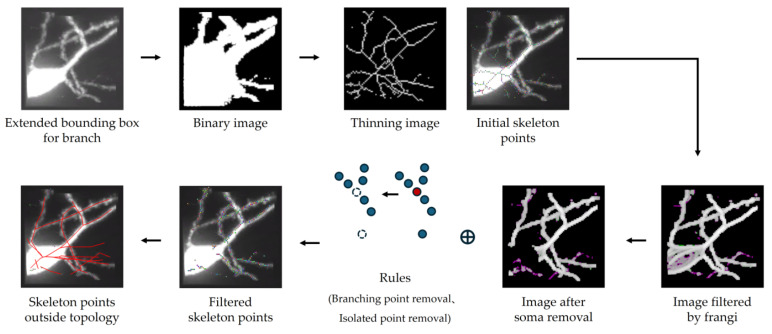
The process of extracting skeleton points for artifacts. Points of different colors represent skeleton points, while red lines depict the neuron reconstruction result within the image block.

In Figure 6, the process of deriving skeleton points for artifacts is demonstrated. In this paper, we introduce an extended bounding box method to better analyze the reconstruction result of each branch. This proposed method retains both the corresponding images of the current branch and its neighbors. First, the process input an image obtained by the extended bounding box method, subsequently performing foreground and background segmentation to acquire a binary graph. For binarization, the process employs a fixed threshold value computed based on the current image attributes [13]. This approach effectively preserves neuronal branches within the image. Next, the thinning algorithm introduced by Palágyi et al. [23] is applied to the binary graph, which is a classic technique for thinning 3D images. After thinning, the non-empty voxels are identified as the initial skeleton points.

Due to the characteristics of neuronal fluorescent labeling, the branches close to the soma are distinctly labeled, often resulting in halos in imaging. These halos can impact the thinning results, leading to the generation of numerous redundant skeleton points. To solve the above problems, this paper filters the images through Frangi filter [24], focusing only on voxels with tubularity. However, it has been observed that the performance of this filter is inadequate near the soma. Consequently, soma removal is conducted based on the reconstructed soma radius. Moreover, if a skeleton point and its adjacent 26 neighborhoods on the processed image are not empty, this point is identified as having tubularity and is retained. Then the process filters the valid skeleton points based on two defined rules: removing branching points to facilitate the extraction of skeleton branching segments, and removing isolated points to reduce image noise interference.

Finally, the skeleton points are corresponding to the reconstruction, and those outside the reconstruction are retained. These points are focal areas for the proposed tool, indicating the potential artifacts’ position. Once the skeleton points of potential artifacts are extracted, they are connected into several undirected skeleton branches through the adjacency matrix. The presence of these skeleton branches represents the likelihood of inaccurately reconstructed branches, implying potential artifacts in the reconstruction.

### 2.5. Branch Confidence Calculation

According to the range of the artifacts’ influence on the branch, the calculation of branch confidence is divided into two categories: global branch confidence calculation and local branch confidence calculation. The global branch confidence calculation is to calculate the confidence of connecting the segments of the parent and child branches in case of global artifacts, including over-tracing and ‘branch–skeleton’ connection errors, and similarly, the local branch confidence calculation is for the local artifacts, which encompass ‘branch–branch’ connection error, branch missing and incomplete tracing.

**Figure 7:**
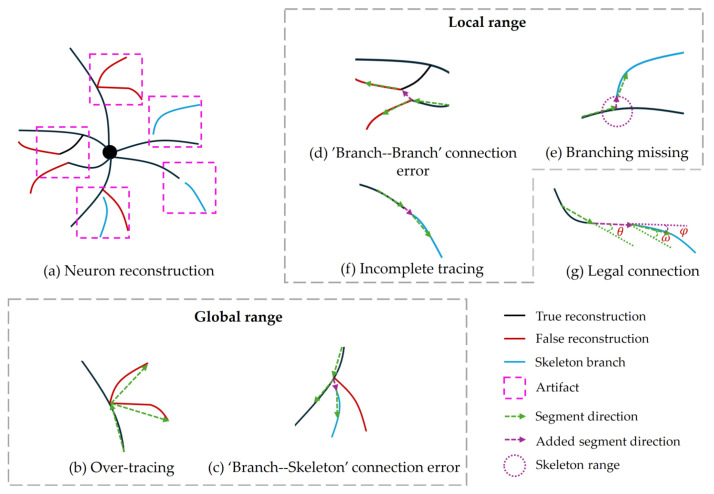
Schematic of reconstruction artifacts.

Figure 7a simulates a case in which a neuron reconstruction contain five kinds of artifacts. In the global range, Figure 7b shows two distinct cases of over-tracing. In the first case, when the angle between the child branching segment and the parent branching segment exceeds 90 degrees, we consider that the child branch lacks the structural feature and advise users to check it. In the second case, the over-tracing is evident when the angle of the branches conforms to the structural feature, then the detection of the artifact relies solely on the image features corresponding to the branching pair. To address this, we propose a branch tubularity calculation: if 70% of the points on the branch have tubularity, the branch is considered to have tubularity. Consequently, when a branch does not have tubularity, it can be reasonably inferred that the branch has an over-tracing artifact. In summary, over-tracing is defined as follows:

(11) θ>90&hasTubularity(CurrentSegment)==false

In this paper, connection errors are categorized into two types depending on the range of influence. In particular, for ‘branch–skeleton’ connection error, as shown in Figure 7c, a branch is incorrectly reconstructed to another location due to interference. As a result, there exists a potential unreconstructed skeleton branch near its parent branching segment. For this type of artifact, we compare the connection probability between the parent branching segment and the skeleton branching segment with that between the parent branching segment and the current branching segment. If the former exhibits a higher value, the current branching has a ‘branch–skeleton’ connection error. The conditions are defined as follows:

(12) P(CurrentSegment|ParentSegment)<P(SkeletonSegment|ParentSegment)

In the local confidence calculation, for the ‘branch–branch’ connection error, depicted in Figure 7d, the branches are in close proximity to each other, resulting in a misconnection of the two branching segments during reconstruction. In this case, the probability of connecting the parent branching segment to other branching segment is higher than that of connecting the parent branching segment to the current branching segment, as defined below:

(13) P(CurrentSegment|ParentSegment)<P(OtherSegment|ParentSegment)

For branch missing, as shown in Figure 7e, it is necessary to consider whether skeleton branches are isolated missing branches. Since a skeleton branch breaks at the branching point, it is possible for the skeleton branch to possess a parent skeleton branch. As for an isolated skeleton branch, the probability of connecting with another skeleton branching segment within the connection range should be lower than that of connecting to the current branching segment. The presence of isolated skeleton branches signifies that the reconstruction result has the artifacts of branches missing. The connection range represents the minimum distance between the skeleton branch and the current branching segment. In conclusion, the criteria for identifying there being a branch missing are defined as follows:

(14) P(CurrentSegment|SkeletonAddedSegment)>P(SkeletonAddedSegment|OtherSekeletonAddedSegment)

(15) Length(SkeletonAddedSegment)>Length(OtherSkeletonAddedSegment)

Finally, for the incomplete tracing detection, it is essential to assess whether adjacent skeleton branches can be legally connected to the current branch. All artifacts’ detection needs to consider the connection legality when identifying potential unreconstructed skeleton branches. In this paper, skeleton branches are considered candidates if they have tubularity and the distance from the endpoint of the current branching segment is less than threshold *D*. The candidate skeleton branches might deviate from the typical growth patterns of neurons when they connect to the current branch, necessitating additional check. As shown in Figure 7g, the growth characteristics of neuronal branches are simulated, and a connection between two neuronal branches is considered legal if the angles of the branching segments satisfy the specified conditions as follows:

(16) θ≤90&φ≤90&ω≤90

when there exists any legal neuron skeleton branch connection, the branch is judged to have incomplete tracing artifact. Based on the various conditions outlined above, branch confidence can be employed for distinguishing among different artifacts. Consequently, only the identified artifacts need to be marked to prompt users to perform the corresponding corrective actions.

## 3. Results

### 3.1. Datasets and Platform

**Figure 8:**
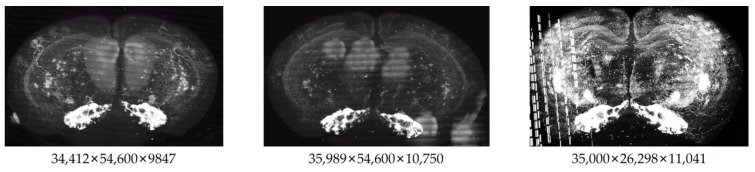
Mouse whole-brain neuronal images.

(1) Whole-Brain Dataset: The proposed tool is to detect the reconstruction artifacts that exist on mouse whole-brain neuronal images, so three different scales of mouse whole-brain neuronal images were chosen to test this tool, with dimensions of 34,412 × 54,600 × 9847, 35,989 × 54,600 × 10,750 and 35,000 × 26,298 × 11,041, all with a resolution of 1 µm × 1 µm × 1 µm, as shown in Figure 8. Ten neurons were arbitrarily chosen from these three large-scale images, and crop image blocks of dimensions of 256 × 256 × 256 with the soma at the center, to create the Whole-Brain dataset.

(2) BigNeuron Dataset: The experimental validation of the tool also can be conducted using the openly available BigNeuron dataset [25], which encompasses neuronal images from various species along with gold standard morphological data. Given the tool’s specific design for mouse neuron analysis, a subset of ten mouse neurons was chosen for testing purposes. In contrast to the whole-brain datasets, this particular dataset predominantly features single neuronal structures with high-quality images at varying resolutions, such as 2048×2048×90, 1288×1280×30, 1324×1252×44 and so on. In order to match the image input dimensions required by this tool, images are resized within the range of 256×256×256.

The tool is developed based on the Vaa3D platform [26], specifically version Vaa3D-x.1.0.8. It is used for 3D visualization and analysis of medical images and interactive operations, and the source code is openly available to support customization by developers. This tool serves as a plug-in for the Vaa3D platform, which is convenient for users to employ directly when reconstructing neurons and plays an auxiliary role.

**Figure 9:**
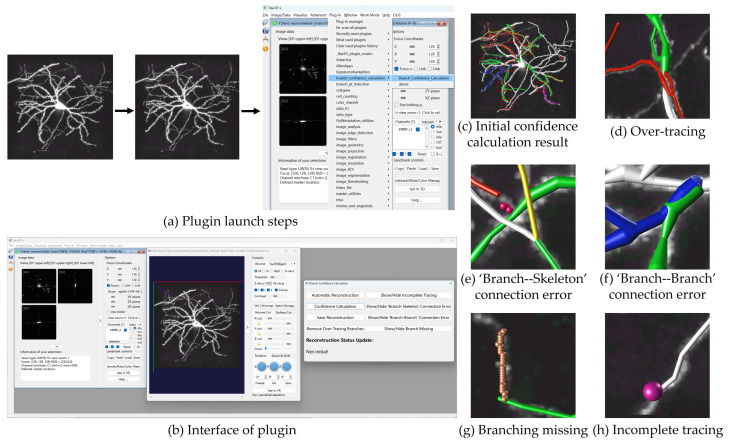
Presentation of system interface and operational results.

Figure 9 displays the interface of this tool along with the presentation of the operational results. First, an image is opened in the Vaa3D platform and the position of soma is marked as the starting position of the reconstruction process. Upon selecting this plugin from Vaa3D’s plugin list, a tool panel for branch confidence calculation will emerge, as shown in Figure 9b. Users can utilize the function buttons on this panel to carry out neuron reconstruction. This paper primarily focuses on the tool’s ability to perform neuron branch confidence calculation for the reconstruction result. Figure 9c–h display the results of the confidence calculation for the automatic reconstruction, and highlight different reconstruction artifacts through different colors. Using the classic APP2 algorithm for automatic reconstruction reveals numerous artifacts, as depicted in Figure 9c. For example, the color red is employed to identify an over-tracing artifact, as demonstrated in Figure 9d, where the red branch forms an angle exceeding 90 degrees with its parent branch. Utilizing yellow to signify the ‘branch–skeleton’ connection error, as exemplified in Figure 9e. The correct position for reconstructing the yellow branch corresponds to the location of the skeleton branch highlighted in the hint. Figure 9f exhibits a blue branch labeled as a ‘branch–branch’ connection error, coinciding with the overlap of the green branch. In Figure 9g, the depiction of a missing branch is indicated by the presence of the skeleton branch, while the green branch signifies the occurrence of the artifact of branch missing. In Figure 9h, the marker hints the position of incomplete tracing, exposing the presence of unfinished voxels at that point. Additionally, Figure 9c features a fuchsine branch, serving as a indicator for users to validate their confidence in the reconstruction. Users can mark a branch in fuchsine when they are convinced that the reconstruction for that branch is absolutely accurate.

The users are able to discern the artifacts in the current branch through the different colors and skeleton branches, allowing them to carry out targeted corrective actions. The reconstruction process using this tool is considered finalized when only white and fuchsine branches are visible in the final result.

### 3.2. Reconstruction Performance

**Figure 10:**
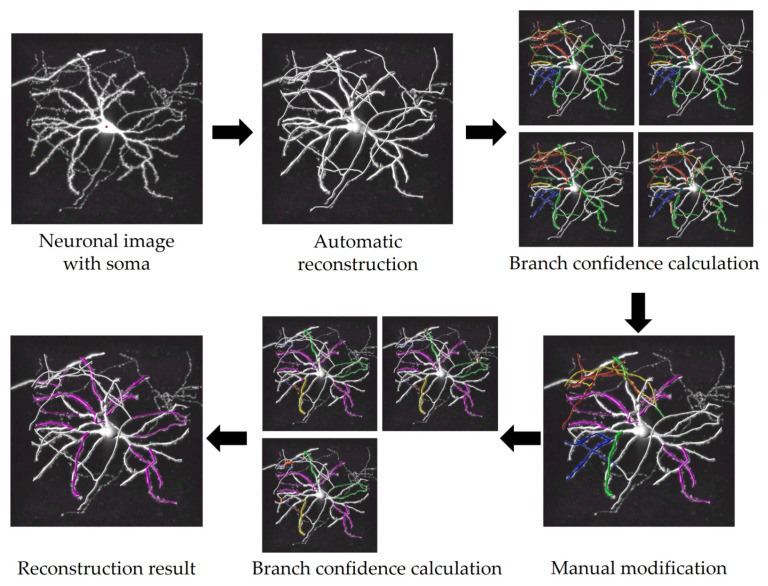
Process of assisting in the complete reconstruction of a neuron using confidence calculation.

The complete process for reconstructing neurons with this tool is depicted in Figure 10. Initially, this tool begins automatic reconstruction upon receiving a neuronal image and soma markers as inputs. Following this, it automatically calculates the confidence of branches in the current reconstruction and marks branches with low confidence based on the rules outlined in Section 3.1. Next, users are prompted to modify the low-confidence branches and save the modified result. After that, another confidence calculation is performed, revealing the persistence of low-confidence branches again. Following further adjustments, all branches transition to either white or fuchsine, signaling the tool’s recognition of the completion of reconstruction. This neuron only carries out two ‘detection–modification’ iterations to achieve a complete reconstruction. In fact, the exact number of iterations required vary for different neurons. This tool calculates branch confidence using parallelization technology, ensuring the detection is to be completed within one minute.

In order to demonstrate the usefulness of this tool in neuron reconstruction, we compared the results reconstructed by manual method, automatic method, automatic followed by manual method, MouseLight method [10] and our method. Similar to our method, the MouseLight method also combines the results of automatic reconstruction with manual reconstruction. By automatically reconstructing neuron fragments and manually connecting branching points, the MouseLight method can achieve efficient axon reconstruction. To facilitate the comparison of neuronal topological morphology, the radii of the reconstruction results were all set to 1.

In Table 1, the results of the neuron reconstruction on the two datasets are evaluated in seven metrics. Among them, Time is used to compare the reconstruction speed of different methods. Precision, Recall, and *F*1-Score are used to judge how well the reconstruction results match the ground truth. The definitions are as follows [27]:

(17) $$Precision\left(R,G\right)=\frac{\left|R\cap G\right|}{\left|R\right|}$$

(18) $$Recall\left(R,G\right)=\frac{\left|R\cap G\right|}{\left|G\right|}$$

(19) $$F1\text{-}Score\left(R,G\right)=2\times \frac{Precision\times Recall}{Precision+Recall}$$

where, *R* denotes the results using different reconstruction methods and *G* denotes the ground truth, which is established through a process of manual reconstruction confirmation. $\left|\cdot \right|$ represents the number of points that satisfy the condition. These metrics are confined within the value range of $\left[0,1\right]$, with a higher numerical value indicating higher reconstruction accuracy.

Additionally, three other metrics, including the entire structure average (ESA), different structure average (DSA), and percentage of different structures (PDS), were employed to evaluate the structural accuracy compared to the ground truth [28]. The lower values for these metrics indicate smaller gaps with ground truths and suggest higher-quality reconstructions.

Analysis of Table 1 indicates that the tool not only accelerates the process of neuron reconstruction but also achieves highly accurate results. In the Whole-Brain dataset, the absence of a gold standard for neuronal morphology led to manual reconstruction results being considered as the ground truth, particularly due to the random selection from the whole-brain images. By analyzing the results of the two datasets, it was observed that automatic reconstruction was the fastest, but less accurate, with the worst F1-Score performance. Notably, automatic reconstruction achieved high accuracy in the BigNeuron dataset, likely due to the dataset’s high level of image quality. The data in Table 1 demonstrate the similarity between our method and the automatic followed by manual method in terms of reconstruction accuracy. This similarity is attributed to both methods prioritizing complete neuron reconstruction. The automatic followed by manual method achieves this by incorporating manual judgment and modification of automatic reconstruction results, while our method relies solely on automatic judgment, leading to differences in time consumption between the two methods. Additionally, significant variations in accuracy can be observed with the MouseLight method. In the Whole-Brain dataset, the MouseLight method achieves higher accuracy levels. This is attributed to the interference from other neuron branches, prompting the method to identify more branching points and segment fragments at locations prone to errors, thereby resulting in higher accuracy in the reconstruction process. Conversely, in the BigNeuron dataset, the lack of human intervention in automatic reconstruction segments significantly impacts the final reconstruction accuracy. Our method consistently achieves accuracy levels close to manual reconstruction, with F1-Scores reaching as high as 93.0% and 92.4%. Consequently, employing this tool to assist reconstruction can achieve accurate neuron reconstruction results.

**Figure 11:**
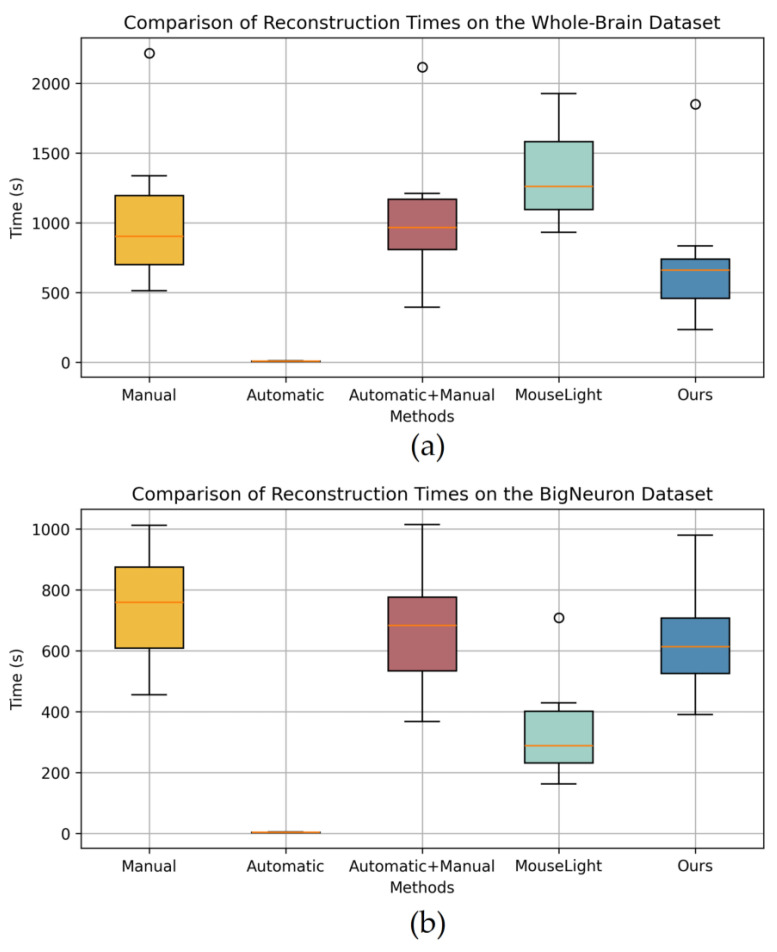
The reconstruction times comparison on the two datasets. (**a**) Comparison of reconstruction times on the Whole-Brain dataset. (**b**) Comparison of reconstruction times on the BigNeuron dataset.

Figure 11 displays the time consumption for reconstruction on the two datasets. It is evident that the time consumed using the proposed tool is lower than the manual method. The automatic method consistently has the shortest processing time, but its reconstruction results with low accuracy cannot be directly utilized. Specifically, the MouseLight method exhibits a longer processing time in Whole-Brain dataset, and even exceeding that of manual reconstruction. This is because the method is primarily applicable to axon reconstruction, and in the whole-brain neuronal images, there are numerous interfering branches around dendrites, leading to the generation of multiple interfering fragments and short fragments. When using this method for whole-brain neuron reconstruction, users must first remove interfering fragments and then connect short fragments, thus requiring a longer processing time. However, in the BigNeuron dataset, due to fewer neuronal signal interferences and higher image quality, longer fragments are generated, requiring only branch point connection operations and resulting in shorter processing times for users.

When reconstructing neurons using the automatic followed by manual method, longer processing times are observed in the Whole-Brain dataset due to the influence of interfering branches, resulting in more reconstruction artifacts in the automatic reconstruction results, requiring user judgment and modifications, which is time-consuming. In contrast, our method demonstrates higher efficiency in reconstructing whole-brain datasets. But there is no significant efficiency advantage over the automatic followed by manual method in the BigNeuron dataset. This is because both methods supplement automatic reconstruction results manually in high-quality images, with the difference lying only in human judgment versus automatic judgment. Consequently, the utilization of the present tool proves effective in reducing the overall time required for neuron reconstruction.

**Figure 12:**
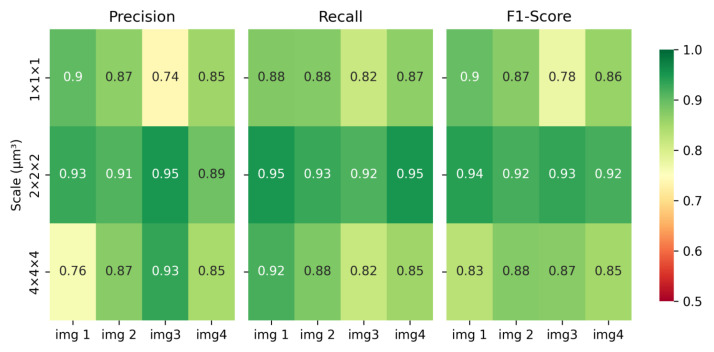
Impact of neuronal image scales on the neuron reconstruction accuracy of our method.

In addition, we also analyze the effect of the magnification of neuronal images on the performance of our method. Four neuronal images from the same mouse brain were chosen from the Whole-Brain dataset, with voxel sizes of 1×1×1 µm^3^, 2×2×2 µm^3^, and 4×4×4 µm^3^, respectively. Neuron reconstruction was performed by the manual method and our method. Figure 12 illustrates the impact of varying image scales on the reconstruction accuracy of our method. Across all scales, our method consistently achieves commendable accuracy, with F1-Scores consistently exceeding 75%. Moreover, our method demonstrates higher accuracy in reconstructing neurons at the 2×2×2 µm^3^ magnification scale compared to the other two scales. At both the 1×1×1 µm^3^ and 2×2×2 µm^3^ scales, intricate details of neuron branches are clearly visible, enabling better feedback for artifact detection and user modification of operations. However, at the 1×1×1 µm^3^ scale, fewer neuron branches are discernible in the image, resulting in some deviations rather than artifacts in reconstruction, which leads to significant fluctuations in accuracy. Similarly, at the 4×4×4 µm^3^ scale, the clarity of branch signals is reduced, resulting in lower reconstruction accuracy compared to the 2×2×2 µm^3^ scale. These findings emphasize the importance of selecting an appropriate scale for reconstructing neuronal images throughout the whole brain.

**Figure 13:**
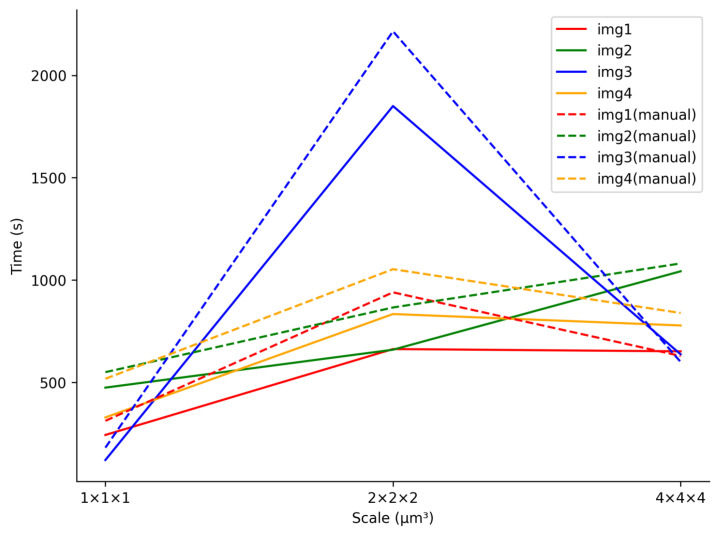
Visualization of reconstruction results on ten different neurons.

Furthermore, Figure 13 illustrates the effect of varying image scales on processing time. As shown in the Figure 13, the reconstruction time using our method increases as the scale rises, attributed to the growing number of neuronal interference branches. Notably, at the 2×2×2 µm^3^ scale, most images require more time for reconstruction because neurons exhibit more fine structure at this scale, requiring additional processing time. And regardless of the scale, the time to reconstruct neurons using our method is often lower than that of the manual method.

**Figure 14:**
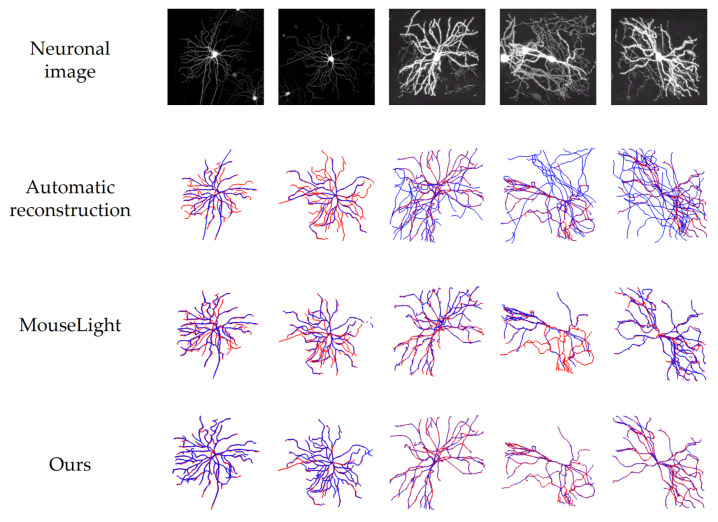
Visualization of reconstruction results on five neuronal images with noise.

Figure 14 visualizes the reconstruction results of five neuronal images with noise. The red part represents the ground truth, the blue part represents the results using different reconstruction methods, and the red and blue overlapped part represents the results matching the ground truth. In Figure 14, it is obvious that the automatic reconstruction results vary in performance across different neuronal images. Many images depict an abundance of blue branches and numerous reconstruction artifacts. On the contrary, when using the MouseLight method to reconstruct neuron images with noise, it is easy to lose branches due to the loss of extracted fragments, resulting in many incomplete red branches in the output. However, when utilizing our method for reconstruction, nearly all branches align closely with the ground truth, resulting in a significantly higher accuracy of reconstruction. This leads us to assert that the proposed tool can provide valuable assistance to users in reconstructing neurons.

## 4. Discussion

By employing the automatic reconstruction algorithm, rapid reconstruction results can be achieved. To mitigate the time-consuming process of manual reconstruction, direct manual adjustments to the automatic reconstruction are feasible. Nevertheless, as evidenced by the outputs depicted in Figure 11, the time invested in this method surpasses the manual method. Moreover, the time required for neuron reconstruction using the MouseLight method is influenced by the quality of the neuronal image. Although high-quality images entail shorter reconstruction times, they often yield lower accuracy in the reconstruction results. Therefore, leveraging this tool to aid users in modifications becomes imperative. Regardless of the complexity of neuron images, this tool is capable of achieving complete reconstruction results similar to manual reconstruction, with accuracy rates exceeding 90% across three performance evaluation metrics. In conclusion, the method of using confidence calculations on branches to detect artifacts in neuron reconstruction is practical.

Presently, automatic reconstruction methods are undergoing continuous updates. However, due to their high specificity, relying only on these algorithms cannot achieve perfect reconstruction for various neuronal images. This tool facilitates a ‘detection–modification’ process on automatic reconstruction results through multiple iterations. Users have the ability to refine the reconstruction by addressing identified artifacts and then proceed to detection following each adjustment, ultimately achieving a highly accurate reconstruction. This strategy does not focus on a particular neuronal image but rather iteratively examines reconstruction results, allowing adaptability to diverse neuronal images by utilizing various automatic reconstruction algorithms. As a result, this efficient process markedly diminishes the labor costs, elevates the reconstruction accuracy, and reduces the reconstruction time.

Actually, branch confidence calculation in neuron reconstruction has been implemented in previous studies. For example, Rivulet2 [29] employs an online confidence calculation which is defined as the proportion of backtracking steps, aimed at removing branches with low confidence. This measure serves to prevent incorrect connections with interfering branches. However, its applicability is limited to the specific automatic reconstruction algorithm used, making it challenging to directly apply to other automatic reconstruction. This paper draws inspiration from the work [20], where connection confidence was introduced to connect various branching segments extracted from an image with high confidence. In contrast, this paper constructs multiple Markov chains for neuron reconstruction, and the branch connection confidence is tailored based on the common features of neuronal branches. A simulation is conducted to assess the confidence associated with connecting branching segments in the presence of various reconstruction artifacts. This tool can be applied to reconstruction results obtained from various automatic reconstruction algorithms and serves as a reference for other studies focusing on neuronal reconstruction artifacts.

The primary focus of this paper is on detecting reconstruction artifacts. This paper outlines the criteria for neuron reconstruction artifacts, and the judgment of some reconstruction artifacts requires considering the features of the extracted skeleton branches. In order to maintain the integrity of foreground voxels within neuronal images, the traditional thinning algorithm is utilized for extracting skeleton points, which needs to be segmented. In this paper, the process of segmenting the neuronal image is simplified by binarizing it based on the intensity features in the image. This simplification might potentially influence the skeleton points extraction process, resulting in redundant skeleton branches and affecting the accuracy of the detection outputs. Recently many neuronal image segmentation methods have been proposed, such as V-Net [30], 3D U-Net Plus [16], and SGSNet [31], which can be used to further optimize this tool.

## 5. Conclusions

In summary, the quest for more efficient manual neuron reconstruction has emerged with the development of various automatic reconstruction algorithms. Nevertheless, the precision of these algorithms heavily relies on the quality of neuronal images, and their outputs are not immediately practical. To tackle this challenge, this paper proposes the integration of a reconstruction artifact detection tool. This tool serves to aid users in modifying and refining the results of automatic reconstruction, ultimately enhancing the practicality and usability of the obtained outputs. In detail, this tool constructs a parent–child branching segments structure by dividing the neuron reconstruction into various Markov chains, subsequently executing connection confidence assessment on these structures. This tool identifies potential unreconstructed skeleton branches in the image, carrying out global and local confidence calculations on these branches. This enables the detection of potential artifacts in the reconstruction, prompting the users to make necessary modifications. Through multiple iterations of automatic detection and users’ intervention, the complete reconstruction of the neuron is achieved.

To assess the tool’s contribution to neuron reconstruction, two neuronal image datasets with varying characteristics were employed: the self-created Whole-Brain dataset and the BigNeuron dataset. When comparing the results obtained through automatic method, automatic followed by manual method, and the MouseLight method to those of our method, and contrasting them with the manual reconstruction results, it is apparent that the tool’s reconstruction aligns more closely with the manual reconstruction and requires less time. In conclusion, this tool can assist the users in realizing a fast and complete neuron reconstruction.

## Figures and Tables

**Table 1:** Performance comparison for neuron reconstruction on the BigNeuron and Whole-Brain datasets.

Dataset	Method	Time (s)	Precision	Recall	F1-Score	ESA	DSA	PDS
Whole-Brain	Manual	1021.656	1.000	1.000	1.000	0.000	0.000	0.000
	Automatic	**8.058**	0.708	0.919	0.764	4.175	12.253	0.187
	Automatic+Manual	1028.262	0.929	**0.937**	0.928	0.819	5.553	0.054
	MouseLight	1348.945	0.802	0.773	0.765	1.915	6.308	0.184
	Ours	704.635	**0.931**	0.930	**0.930**	**0.753**	**4.948**	**0.051**
BigNeuron	Manual	743.366	0.967	**0.907**	**0.936**	**0.934**	4.411	**0.049**
	Automatic	**3.560**	0.968	0.346	0.499	4.918	8.290	0.313
	Automatic+Manual	669.420	0.950	0.850	0.896	1.066	4.104	0.080
	MouseLight	332.586	0.834	0.561	0.641	2.637	5.687	0.268
	Ours	620.583	**0.969**	0.883	0.924	0.957	**4.074**	0.057

Note: Automatic+Manual means the automatic followed by manual method. Bold values indicate superior performance in the corresponding metric. In the Whole-Brain dataset, the Manual method is established as the ground truth without comparative analysis.

## Data Availability

All data can be provided upon request to the corresponding author due to the large volume of data.

## References

[1] Petrella J.R., Coleman R.E., Doraiswamy P.M. (2003). Neuroimaging and early diagnosis of Alzheimer disease: A look to the future. Radiology.

[2] Giorgio A., De Stefano N. (2013). Clinical use of brain volumetry. J. Magn. Reson. Imaging.

[3] Kulkarni V.A., Firestein B.L. (2012). The dendritic tree and brain disorders. Mol. Cell. Neurosci..

[4] Gong H., Xu D., Yuan J., Li X., Guo C., Peng J., Li Y., Schwarz L.A., Li A., Hu B. (2016). High-throughput dual-colour precision imaging for brain-wide connectome with cytoarchitectonic landmarks at the cellular level. Nat. Commun..

[5] Wang X., Xiong H., Liu Y., Yang T., Li A., Huang F., Yin F., Su L., Liu L., Li N. (2021). Chemical sectioning fluorescence tomography: High-throughput, high-contrast, multicolor, whole-brain imaging at subcellular resolution. Cell Rep..

[6] Dodt H.U., Leischner U., Schierloh A., Jährling N., Mauch C.P., Deininger K., Deussing J.M., Eder M., Zieglgänsberger W., Becker K. (2007). Ultramicroscopy: Three-dimensional visualization of neuronal networks in the whole mouse brain. Nat. Methods.

[7] Ahrens M.B., Orger M.B., Robson D.N., Li J.M., Keller P.J. (2013). Whole-brain functional imaging at cellular resolution using light-sheet microscopy. Nat. Methods.

[8] Glaser J.R., Glaser E.M. (1990). Neuron imaging with Neurolucida—A PC-based system for image combining microscopy. Comput. Med. Imaging Graph..

[9] Peng H., Bria A., Zhou Z., Iannello G., Long F. (2014). Extensible visualization and analysis for multidimensional images using Vaa3D. Nat. Protoc..

[10] Winnubst J., Bas E., Ferreira T.A., Wu Z., Economo M.N., Edson P., Arthur B.J., Bruns C., Rokicki K., Schauder D. (2019). Reconstruction of 1,000 projection neurons reveals new cell types and organization of long-range connectivity in the mouse brain. Cell.

[11] Economo M.N., Clack N.G., Lavis L.D., Gerfen C.R., Svoboda K., Myers E.W., Chandrashekar J. (2016). A platform for brain-wide imaging and reconstruction of individual neurons. eLife.

[12] Peng H., Meijering E., Ascoli G.A. (2015). From diadem to bigneuron. Neuroinformatics.

[13] Xiao H., Peng H. (2013). APP2: Automatic tracing of 3D neuron morphology based on hierarchical pruning of a gray-weighted image distance-tree. Bioinformatics.

[14] Ming X., Li A., Wu J., Yan C., Ding W., Gong H., Zeng S., Liu Q. (2013). Rapid reconstruction of 3D neuronal morphology from light microscopy images with augmented rayburst sampling. PLoS ONE.

[15] Wang C.W., Lee Y.C., Pradana H., Zhou Z., Peng H. (2017). Ensemble neuron tracer for 3D neuron reconstruction. Neuroinformatics.

[16] Li Q., Shen L. (2019). 3D neuron reconstruction in tangled neuronal image with deep networks. IEEE Trans. Med. Imaging.

[17] Zhao J., Chen X., Xiong Z., Liu D., Zeng J., Xie C., Zhang Y., Zha Z.J., Bi G., Wu F. (2020). Neuronal population reconstruction from ultra-scale optical microscopy images via progressive learning. IEEE Trans. Med. Imaging.

[18] Chen W., Liu M., Du H., Radojević M., Wang Y., Meijering E. (2021). Deep-learning-based automated neuron reconstruction from 3D microscopy images using synthetic training images. IEEE Trans. Med. Imaging.

[19] Radojević M., Meijering E. (2017). Automated neuron tracing using probability hypothesis density filtering. Bioinformatics.

[20] Athey T.L., Tward D.J., Mueller U., Vogelstein J.T., Miller M.I. (2022). Hidden Markov modeling for maximum probability neuron reconstruction. Commun. Biol..

[21] Li S., Quan T., Zhou H., Yin F., Li A., Fu L., Luo Q., Gong H., Zeng S. (2019). Identifying weak signals in inhomogeneous neuronal images for large-scale tracing of sparsely distributed neurites. Neuroinformatics.

[22] Liu Y., Zhong Y., Zhao X., Liu L., Ding L., Peng H. (2023). Tracing weak neuron fibers. Bioinformatics.

[23] Palágyi K., Kuba A. (1999). A parallel 3D 12-subiteration thinning algorithm. Graph. Model. Image Process..

[24] Radojević M., Meijering E. (2019). Automated neuron reconstruction from 3D fluorescence microscopy images using sequential Monte Carlo estimation. Neuroinformatics.

[25] Peng H., Hawrylycz M., Roskams J., Hill S., Spruston N., Meijering E., Ascoli G.A. (2015). BigNeuron: Large-scale 3D neuron reconstruction from optical microscopy images. Neuron.

[26] Peng H., Ruan Z., Long F., Simpson J.H., Myers E.W. (2010). V3D enables real-time 3D visualization and quantitative analysis of large-scale biological image data sets. Nat. Biotechnol..

[27] Quan T., Zhou H., Li J., Li S., Li A., Li Y., Lv X., Luo Q., Gong H., Zeng S. (2016). NeuroGPS-Tree: Automatic reconstruction of large-scale neuronal populations with dense neurites. Nat. Methods.

[28] Peng H., Ruan Z., Atasoy D., Sternson S. (2010). Automatic reconstruction of 3D neuron structures using a graph-augmented deformable model. Bioinformatics.

[29] Liu S., Zhang D., Song Y., Peng H., Cai W. (2018). Automated 3-D neuron tracing with precise branch erasing and confidence controlled back tracking. IEEE Trans. Med. Imaging.

[30] Liu M., Luo H., Tan Y., Wang X., Chen W. (2018). Improved V-Net Based Image Segmentation for 3D Neuron Reconstruction. Proceedings of the 2018 IEEE International Conference on Bioinformatics and Biomedicine (BIBM).

[31] Yang B., Liu M., Wang Y., Zhang K., Meijering E. (2021). Structure-guided segmentation for 3D neuron reconstruction. IEEE Trans. Med. Imaging.

